# Impaired Brain-Heart Relation in Patients With Methamphetamine Use Disorder During VR Induction of Drug Cue Reactivity

**DOI:** 10.1109/JTEHM.2022.3206333

**Published:** 2022-09-14

**Authors:** Chun-Chuan Chen, Chia-Ru Chung, Meng-Chang Tsai, Eric Hsiao-Kuang Wu, Po-Ru Chiu, Po-Yi Tsai, Shih-Ching Yeh

**Affiliations:** Department of Biomedical Sciences and EngineeringNational Central University 34911 Taoyuan City 320317 Taiwan; Computer Science and Information Engineering DepartmentNational Central University 34911 Taoyuan City 320317 Taiwan; Department of PsychiatryKaohsiung Chang Gung Memorial Hospital Kaohsiung City 83301 Taiwan; Department of PsychiatryChang Gung University College of Medicine 71589 Kaohsiung City 83301 Taiwan; Department of Physical Medicine and RehabilitationTaipei Veterans General Hospital 46615 Taipei 112201 Taiwan

**Keywords:** Neuronal abnormalities, methamphetamine use disorder (MUD), virtual reality (VR), EEG, heart rate variability (HRV), impaired brain-heart relation

## Abstract

Methamphetamine use disorder (MUD) is an illness associated with severe health consequences. Virtual reality (VR) is used to induce the drug-cue reactivity and significant EEG and ECG abnormalities were found in MUD patients. However, whether a link exists between EEG and ECG abnormalities in patients with MUD during exposure to drug cues remains unknown. This is important from the therapeutic viewpoint because different treatment strategies may be applied when EEG abnormalities and ECG irregularities are complications of MUD. We designed a VR system with drug cues and EEG and ECG were recorded during VR exposure. Sixteen patients with MUD and sixteen healthy subjects were recruited. Statistical tests and Pearson correlation were employed to analyze the EEG and ECG. The results showed that, during VR induction, the patients with MUD but not healthy controls showed significant $\alpha $ and $\beta $ power increases when the stimulus materials were most intense. This finding indicated that the stimuli are indiscriminate to healthy controls but meaningful to patients with MUD. Five heart rate variability (HRV) indexes significantly differed between patients and controls, suggesting abnormalities in the reaction of patient’s autonomic nervous system. Importantly, significant relations between EEG and HRV indexes changes were only identified in the controls, but not in MUD patients, signifying a disruption of brain-heart relations in patients. Our findings of stimulus-specific EEG changes and the impaired brain-heart relations in patients with MUD shed light on the understanding of drug-cue reactivity and may be used to design diagnostic and/or therapeutic strategies for MUD.

## I. Introduction

Methamphetamine (METH), a highly addictive psychostimulant, is the second most widely used class of illicit drugs worldwide and can cause irreversible damage of brain cells [1]. Chronic METH users exhibit neurological (structural) and psychiatric (functional) abnormalities. METH use disorder (MUD) is considered a brain disease [2]. Drug-cue reactivity, including mental (e.g., craving and attention bias), psychophysiological (e.g., heart rate, skin conductance, and brain activity), and behavioral (e.g., drug-seeking) responses, is evoked to study the underlying mechanisms in patients with MUD [3], [4]. Experimental paradigms have been developed for eliciting drug-cue reactivity by presenting drug-related stimuli to drug users, including uni-sensory (e.g., visual, auditory, or olfactory) and multi-sensory (e.g., audiovisual and sensory) stimuli associated with previous drug use. Many factors can influence drug-cue reactivity, including cue characteristics, individual factors and contextual factors [5]. Traditionally, one or several drug-related cues, such as pictures, photographs, videos and objects, are presented to the subjects, who are instructed to either passively experience the drug cues or actively respond to these stimuli [6]. Due to a lack of complex difficulty in establishing standardization, applications of traditional cue-reactivity studies are limited [6]. Compared with traditional drug-cue reactivity paradigms, VR advantages include immersion, flexibility and ease of integration into clinical settings and has been applied in the field of addictive disorders [4], [6], [7]. Specifically, complex cues embedded in VR have been demonstrated to induce a sharper and more rapid craving increase [8], [9], [10] and stronger attentional bias toward substance-related cues [11], [12], [13], [14] in several substance abuse disorders [see also [6] for a review). Indeed, VR can efficiently induce drug-cue reactivity and could be used to address several problems related to substance abuse, from craving assessment to active craving treatment using cue-exposure therapy or cognitive behavioral therapy [6]. Using VR in patients with MUD has been shown to elicit significant frequency-specific neuronal abnormalities [15], [16], [17] and ECG changes during the induction of drug craving [18], [19]. However, whether a link exists between neuronal and ECG changes in patients with MUD during drug-cue exposure has not yet been explored. Furthermore, whether different intensities/contents of drug cues lead to different neuronal states and ECG changes remains unclear.

In this study, we used an integrated VR system with gradual intensities of drug cues to investigate whether there is any relation between EEG and ECG changes in patients with MUD. In addition, we examined the EEG activities and ECG variabilities that may reflect different stimulations to validate the efficacy of the VR system.

This paper is organized as follows: Section II describes the updated findings regarding METH, EEG and ECG in the literature. Section III describes the details of the stimulation materials and the VR system used, along with the experiment procedures, data analysis, and statistical tests. Section IV presents the experimental results. The discussion and conclusions are summarized in Section V.

## II. Related Work

MUD is gaining attention as METH leads to considerable behavioral problems (see [20], [21]] for review) and is the primary culprit in overdose deaths [22]. Chronic METH users develop neurological abnormalities [2], engaging the areas in the mesocorticolimbic reward circuit and executive control circuit [20], [23], [24]. EEG provides real-time measurements of brain activities in response to drug cues with very high temporal resolution. Features extracted from EEG can serve as the endophenotypes to discover the biological underpinnings of neurocognitive and neurophysiological impairments in substance abuse disorder [25]. In general, brain activities measured with EEG can be divided into different rhythms, including $\delta $ (0.1– 4 Hz), $\ominus $ (4–8Hz), $\alpha $ (8–15 Hz), $\beta $ (15–30 Hz) and $\gamma $ (30-48 Hz) frequencies [26] and different oscillatory signals may have different functional roles. For instance, $\ominus $ oscillations were reported to be related with impulse control and action monitoring, $\alpha $ power involved in suppression of task-irrelevant responses, $\beta $ activities were engaged in active concentration and motor preparation, and $\gamma $ activities were associated with perceptual processing, attention and memory [27]. Therefore, it was suggested that investigation of the above four frequency bands should provide insights for the functional changes after abuse of METH [17], as patients with MUD exhibit impairments in working memory, error monitoring and attention. Tan et al. employed drug-related stimuli in a VR environment to elicit brain drug cue reactivity in patients with MUD and found that the $\gamma $ (30–48 Hz) activity in the frontal areas decreased during VR induction of drug craving when compared to neutral cues [15]. In 2020, Ding et al. employed machine- learning methods to distinguish patients with MUD from healthy subjects using frequency-specific EEG features during VR induction of drug-cue reactivity. They reported that patients with MUD showed higher $\gamma $ power and smaller EEG power in lower frequency bands, including $\delta $ and $\alpha $ bands, during VR induction [16]. In 2021, we reported that patients with MUD still exhibited abnormalities after VR induction in $\ominus $, $\alpha $ and $\gamma $ power changes during a resting state.

Heart rate is important to maintain a normal physiological state. Many factors, such as emotions, exercise and diseases, can affect the heart rate, leading to variabilities in heart rate. Heart rate variability (HRV) analysis using ECG signals is considered an effective way to measure the cardiac autonomic modulation of the autonomic nervous system (ANS) and is wildly applied in different diseases [28], [29], [30], [31]. METH use causes sympathomimetic amines and cardiac complications are often seen in MUD patients, including hypertension, aortic dissection, acute coronary syndromes and pulmonary arterial hypertension [32], [33]. It has been shown that the changes of HRV indexes on time, frequency and nonlinear domains in MUD patients differ significantly from healthy controls [18], [19]. These HRV changes in patients positively correlated with the level of METH craving in MUD patients [19], [34].

In summary, abuse of METH may result in abnormalities in frequency-specific regional neuronal activity and in HRV indexes in response to drug-related cues. These findings raise the question of whether EEG abnormalities and ECG irregularities are comorbidity or complications in patients with MUD. This is important from the therapeutic viewpoint because different treatment strategies may be used for comorbidities or complications.

## III. Material and Method

### A. System Equipment

In this study, we used an intelligent VR system integrated with EEG and multiple stimuli for reliably inducing intensity-specific drug cue-related changes. This system has been used in our previous works [17], [18]. HTC Vive Pro Eye®with an embedded headset and VR controllers were used to create an immersive three-dimensional visual and interactive experience. In addition, a wearable device by ScentRealmTM X-Scent3.0®was employed to provide odor stimulations of beer, cigarette, METH, and a karaoke room, with controllable density and stimulation frequency. Furthermore, the VR system was integrated with various sensors that measured the physiological signals of EEG data (BrainVision V-amp), ECG (Thought Tech TM), galvanic skin response (GSR), and eye movements using Unity 3D, therefore, data from these devices would coordinately output with a time stamp for synchronization. Before the start of the experiment, we calibrated the position of the participant to be in the center of the VR system. The participant was seated comfortably with VR controllers in their right hand to interact with the virtual environment. Through HTC Vive Pro Eye®, a variety of visual and acoustic stimulations, such as beer, smoke, drugs, music, TV, and enticing conservations with nonplayer characters (NPCs), were delivered to the participant.

### B. VR Scenario: Gradual Intensities of Drug Cues

**FIGURE 1.**
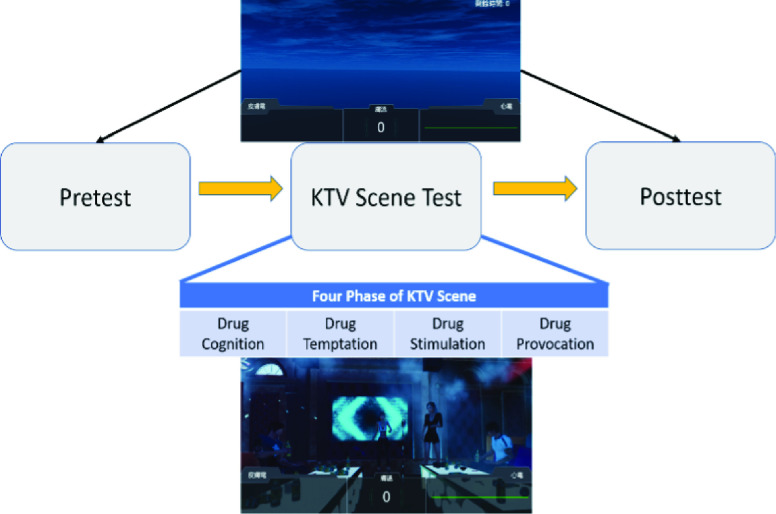
Experimental procedure and the virtual scenes seen by the subject.

For better manipulation of drug cues, we used Unity3D to create a virtual scene with gradual intensities of drug cues. The virtual scene is a karaoke environment (Fig. 1) that mimics the environment where patients with MUD used METH. Four stimulation stages were designed to gradually enhance the drug cue-related reactivity:

1) Stage I: Drug Cognition – Images of several drugs, including FM2, heroin, ketamine, amphetamine, ecstasy, cannabis, and METH, were randomly presented to participants with a refresh rate of six seconds through the headset, along with a voice prompt, to enhance drug memory recall.
2) Stage II: Drug Temptation – This stage aims to evoke the drug craving by providing an atmosphere and situations that usually elicit the desire for drugs in MUD patients. In this stage, NPCs take part in typical karaoke room activities, such as smoking, drinking, chatting, dancing and singing. Finally, an NPC asks the participant to join them.
3) Stage III: Drug Stimulation – To further increase temptation, this stage stimulates the mirror neuron system of the participant by showing an animation of an NPC taking METH. An intense smell of METH accompanies this action as well. Furthermore, conversations between NPCs revolve around METH intake to further entice the participant.
4) Stage IV: Drug Provocation – This stage attempts to convert the drug desire into action and punishment. The NPCs attempt to verbally persuade the participant to fulfill their desire and ask them to decide whether they want to try or reject. This is to mimic the struggle in the patient’s mind. At the same time, police sirens are played to act as a strong deterrent to the patient with MUD.

Total duration of the VR induction’s four stages is 12 minutes. To maintain participant engagement, participants are asked to press a button within 3 seconds whenever they hear the word “you” spoken by the voice prompt.

### C. Subjects

Sixteen MUD patients (11 males, 5 females) were recruited from Chang Gung Memorial Hospital. The inclusion criteria for participants were as follows: (1) aged 20–65 years, and (2) diagnosed as having MUD based on the Diagnostic and Statistical Manual of Mental Disorders, Fifth Edition (DSM-V) by a board-certified psychiatrist. Exclusion criteria were a history of major psychiatric illness, physical illnesses in stable condition, and heart disease. A group of 16 healthy subjects (12 males, 4 female) without mental or physical diseases were also recruited for comparison. Subjects in the healthy group were not taking any prescription medication. The study was performed at Kaohsiung Chang Gung Memorial Hospital and was approved by the Institutional Review Board of Kaohsiung Chang Gung Memorial Hospital (201901912A3) in accordance with the Declaration of Helsinki and Good Clinical Practice guidelines. All participants signed an informed consent after receiving an explanation of the study.

### D. EEG Acquisition and Processing

Electrophysiological data of EEG from five channels (C3, C4, Cz, Pz and FCz) and ECG signals were recorded with the sampling rate of 512 Hz during the VR experiment. Horizontal and vertical EOG were also recorded for artifact removal. To maintain their attention throughout the experiment, subjects were instructed to press a button using their right index finger whenever they heard the word ‘you.’ These data were bandpass filtered (2-56 Hz) offline using Butterworth filter in SPM12 (Wellcome Trust Centre for Neuroimaging, https://www.fil.ion.ucl.ac.uk /spm/) to remove head motion (< 1Hz) and power line (60Hz) artefacts, and EOG contaminations were removed from the EEG data using a fully automated method [35]. The EOG-removed data were then transformed into the time-frequency domain from 4 to 48Hz using Morlet wavelet (wavelet number: 5). The spectral densities at each channel were epoched according to the stimulus stages and averaged into four frequency bands of interest, including the $\ominus $ (4-7 Hz), $\alpha $ (8-14 Hz), $\beta $ (15-24 Hz) and $\gamma $ (25-48 Hz) bands. Finally, the frequency-specific spectrum were averaged over time points to obtain the stage-specific data. We used the first-minute averaged data before VR exposure of each subject as the individual baseline and computed the relative frequency-specific power change ratio (PCR) as follows:

\begin{equation*} \mathrm{PCR}=\big(\mathrm{P}-\text { Pbaseline }\big] / \text { Pbaseline } \times 100 \% \end{equation*}

where P is the phase- and frequency-specific EEG power and Pbaseline is the individual frequency-specific baselines.

### E. ECG Data Processing

The raw ECG data were first down-sampled to 256 Hz and filtered with a band-stop filter of 60 Hz to remove the power line artifacts. Fifteen HRV indexes for each participant were extracted from the filtered ECG data using the Kubios HRV Analysis Software package [36]. These HRV indexes contain eight time-domain related parameters, five frequency-specific and two nonlinear parameters (see Supplementary Table 1 for the list of HRV indexes). For a better estimate of the HRV indexes with enough time points, we combined two stages of ECG data, resulting in only two stage-specific ECG parameters. For correlation analysis of EEG and ECG, we merged the EEG data of two stages.

**TABLE 1:** Demographic Characteristics and Clinical Scales

Variable	Patients ( $n=16$)	Controls ( $n=16$)	Significance ( $P$-value)
Age, years	44.2±8.5	33.6±9.5	0.53
Male/ Female	11 /5	12 /4	
BMI	25.8±4.3	23.4±4.1	0.12

### F. Statistical Analyses

The PCR differences of EEG at the five channels were statistically tested using analysis of variance (ANOVA) for within- and between-groups factors using SPSS AMOS 21.0. The HRV indexes were also entered using ANOVA with two factors: (1) group with two levels (patients vs. controls), and (2) HRV indexes with 15 variables. A post hoc t-test with control of false discovery rate was applied to further identify the significant pairwise difference among the factors between groups. Paired t-test was employed to examine the within-group PCR differences in both groups. Pearson’s correlation was used to establish the relations between PCRs and HRV indexes. For the demographic characteristics, two sample t-tests were used to test for differences between the two groups. The significant level was set at $\text{p} < 0.05$ after correction.

## IV. Result

### A. Demographic Characteristics and Clinical Scales

Table 1 presents the statistical results of the demographic characteristics of the patients and healthy subjects. The mean age of the healthy group was younger, but the age difference between the two groups was not significant (*p = 0.53*). The two groups exhibited similar BMI (*p = 0.12*).

### B. Within Group Power Differences Induced By VR

To validate the efficacy of this VR system on eliciting brain changes, we first investigated the within-group effects of PCRs in EEG power when compared with the baseline (Table 2). For the patient group, the VR experiment robustly induced $\alpha $ power decreasing of all channels during Stage I and II. These $\alpha $ decreases extended to stage III for all channels except Pz in the patient group. The $\beta $ power were suppressed in Stage I at Cz and FCz, in Stage II at all channels except Pz, and in Stage III at C4, Cz, and FCz. The $\ominus $ PCR decreased in Stage I at Cz and Stage II at FCz. The $\gamma $ PCR were decreased only at Cz in Stage I and II. No PCRs were significant in Stage IV in patients. For the healthy subjects, the most significant decreases were the $\gamma $ PCRs at all channels in Stage III and at Cz, Pz, FCz in Stage IV (Table 2). The $\beta $ power were suppressed in Stage IV at C3, Pz and Cz.

**TABLE 2:** Significant Frequency-Specific Within Group PCRs

EEG feature	Patient Group				Healthy Group			
	STAGE I	STAGE II	STAGE III	STAGE IV	STAGE I	STAGE II	STAGE III	STAGE IV
θ C3	−10.4 ± 42.9	−12.3 ± 44.4	−11.6 ± 45.8	8.6 ± 1.6	−12.3 ± 29.5	−8.7 ± 26.7	−8.8±27.9	−10.2±18.7
θ C4	−12.0 ± 42.6	−15.6 ± 44.9	−12.7 ± 50.2	6.4 ± 8.8	−13.2 ± 32.5	−10.0 ±27.9	−9.0±28.9	−10.2±20.5
θ Cz	−21.1 $\pm 27.5\ast $	−18.8 ± 30.4	−11.4 ± 39.1	2.5 ± 2.0	−9.6 ±21.8	−5.2 ± 20.4	−8.0±23.9	−8.6±13.4
θ Pz	−14.2 ± 45.8	−17.3 ± 46.2	9.0 ± 85.3	24.8 ± 3.0	−11.2 ± 26.1	−7.0±24.4	−8.6±26.2	−9.6±16.3
θ FCz	−18.1± 40.9	−25.9 $\pm 35.4\ast $	−16.7 ± 43.6	0.5 ± 5.5	−13.8 ± 34.9	−11.1 ± 28.6	−9.5±29.3	−9.8±22.2
$\alpha $C3	−25.3 $\pm 33.1\ast \ast $	−28.5 $\pm 35.7\ast $	−22.3 $\pm 40.5\ast $	19.7 ± 15.8	−16.4 ± 39.9	−21.3 ±39.6	−15.4±33.9	−16.1±22.7
$\alpha $C4	−22.9 $\pm 30.4\ast $	−27.6 $\pm 33.8\ast \ast $	−25.4 $\pm 34.4\ast $	19.8 ± 26.4	−17.4± 41.6	−20.6 ±27.4	−14.7±36.0	−16.1±24.6
$\alpha $Cz	−27.1 $\pm 34.3\ast $	−33.1 $\pm 31.6\ast \ast $	−30.4 $\pm 32.4\ast \ast $	2.3 ± −8.0	−13.3 ± 33.1	−23.6 ± 39.0	−18.2±30.1	−15.1±18.0
$\alpha $Pz	−25.5 $\pm 36.5\ast $	−31.5 $\pm 40.6\ast \ast $	−3.3 ± 91.6	37.8 ± 29.1	−15.1 ± 37.5	−26.1±59.0	−16.4±31.9	−15.8±20.5
$\alpha $FCz	−30.3 $\pm 33.1\ast \ast $	−38.9 $\pm 28.8\ast \ast $	-32.7 $\pm 31.8\ast \ast $	11.4 ± 22.8	−18.1 ± 43.3	−22.2 ±16.6	−13.9±37.5	−5.9±25.9
$\beta $C3	−15.3 ± 35.3	−19.2 $\pm 37.1\ast $	−16.1 ± 40.2	19.9 ± 2.5	−23.1 ±39.5	−7.1 ±28.8	−17.7±26.5	−12.9 $\pm 21.6\ast $
$\beta $C4	−16.3 ± 35.1	−22.7 $\pm 36.0\ast $	−21.0 $\pm 37.5\ast $	15.6 ± 2.0	−24.3 ±40.2	−16.8 ±18.7	−18.6±27.9	−13.2±23.7
$\beta $Cz	−28.3 $\pm 27.0\ast \ast $	−34.7 $\pm 25.3\ast \ast $	−21.5 $\pm 37.8\ast $	11.3 ± 4.6	−18.5 ± 32.4	−18.1 ±24.6	−14.8±22.5	−11.0 $\pm 15.4\ast $
$\beta $Pz	−15.9 ± 41.0	−25.7 ± 47.4	−7.6 ± 74.8	35.1 ± 19.9	−21.3 ±37.7	−17.2 ±18.9	−16.5±24.6	−12.2 $\pm 18.8\ast $
$\beta $FCz	−22.7 $\pm 36.3\ast $	−32.4 $\pm 33.2\ast \ast $	−26.0 $\pm 37.0\ast $	10.5 ± 6.2	−25.1 ±41.8	−16.1 ±18.0	−19.4±28.5	−13.2±25.1
$\gamma $C3	4.0 ± 41.2	−1.9 ± 40.5	−2.5 ± 45.2	18.2 ± 33.0	−23.8 ± 53.4	−18.7 ±24.8	−20.9 $\pm 28.1\ast $	−14.0±25.0
$\gamma $C4	−1.4 ± 40.7	−10.4 ± 38.3	−11.1 ± 41.3	9.3 ± 9.6	−25.1 ± 49.1	−18.6 ±25.4	−21.6 $\pm 28.9\ast $	−14.4±27.5
$\gamma $Cz	−17.1 $\pm 22.6\ast $	−24.8 $\pm 29.0\ast $	−10.9 ± 45.5	6.8 ± 4.5	−19.3 ±27.1	−18.4 ±22.8	−18.3 $\pm 25.4\ast $	−12.4 $\pm 18.8\ast $
$\gamma $Pz	0.9 ± 49.1	2.8 ± 75.4	−0.7 ± 66.3	29.9 ± 7.1	−21.8 ± 31.5	−18.6 ±23.9	−19.8 $\pm 26.9\ast $	−13.4 $\pm 22.0\ast $
$\gamma $FCz	−5.4 ± 44.2	−19.7 ± 39.7	−14.7 ± 44.5	6.0 ± 2.3	−25.7 ± 34.9	−18.6±25.4	−22.2 $\pm 29.1\ast $	−14.7±29.6

$\ast $: $\text{P} < 0.05$ after correction

### C. Stage-Specific Differences in EEG Power During VR Induction

**FIGURE 2.**
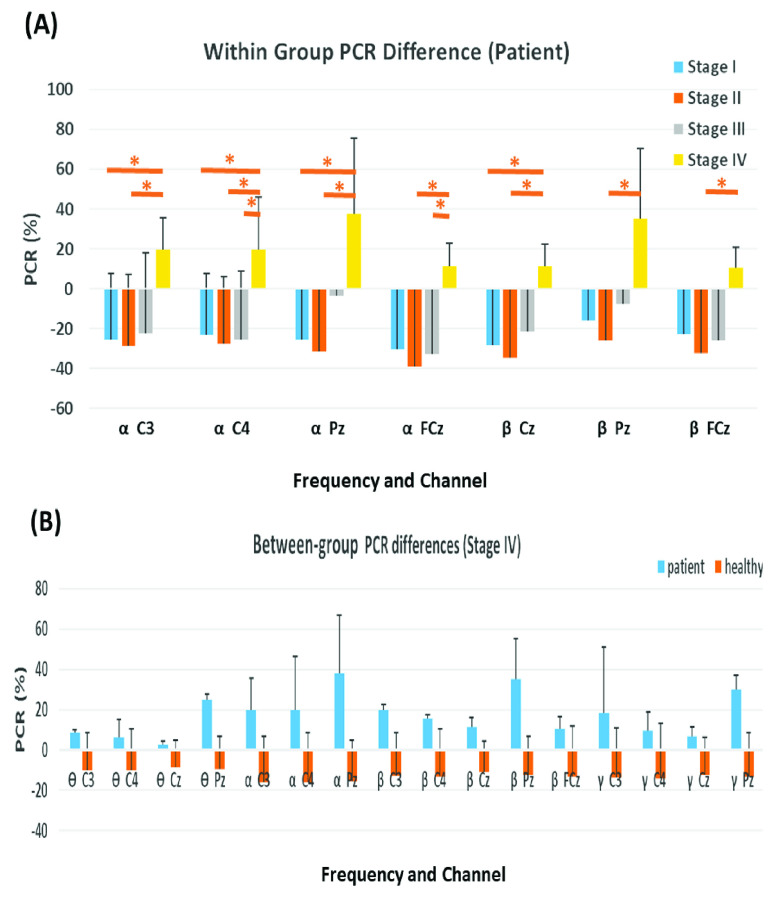
(A) Significant stage-specific PCR changes of the patient group. The asterisks indicate significant differences between stages after correction for multiple comparisons ( $\text{P} < 0.05$); (B) Significant between-group PCR changes in stage IV.

Figure 2A shows the significant stage-specific differences in EEG power of the post hoc tests for the patient group. At first glance, the PCRs reversely become positive in Stage IV whereas were negative in other stages (Figure 2A). In particular, significant enhancements in $\alpha $ and $\beta $ PCR can be seen in Stage IV over the midline areas of FCz, Cz and Pz and the primary motor areas of C3 and C4. The $\alpha $ power in Stage IV were significantly greater than that in Stage I at C3, C4 and Pz, in Stage II at C3, C4, FCz and Pz, and in Stage III at C4 and FCz. The $\beta $ oscillations in Stage IV were significantly larger than that in stage IV at Cz, and in Stage II at Cz, Pz and FCz. There were no significant stage-specific differences in $\ominus $ and $\gamma $ power in the patients. Importantly, for the healthy subjects, no stage-specific differences in EEG power during VR Induction were identified. Regarding the between-group PRC differences, significant PCR differences between patients and controls were found only in Stage IV, at all frequencies and channels tested, except $\ominus $, $\alpha $, and $\gamma $ at FCz and $\alpha $ at Cz (Figure 2B).

### D. HRV Differences Between Patients and Controls During VR Induction

Table 3 shows the significant HRV differences between patients and controls during VR induction. Among 15 ECG features being tested, seven features significantly differ between the two groups, including MRRI, SDNN, BNN20,BPNN20, BVLF, BLF, BSD2. Specifically, NN20, PNN20 and SDNN were significantly smaller throughout the VR experiment in MUD patients compared with the healthy controls. MRRI and LF during Stage I and II and VLF during Stage III and IV were significantly smaller in MUD patients. Only SD2 during Stage I and II was significantly greater in the patient group.

**TABLE 3:** Significant HRV Differences Between Patients and Controls

	Patient	Normal		
ECG Feature	Average ± SD	Average ± SD	t value	p
MRRI Stage I and II	700.7 ± 119.3	775.46 ± 86.3	−3.2	< 0.001
SDNN Stage I and II	29.2 ± 14.5	48.1 ± 18.3	−4.1	< 0.001
NN20 Stage I and II	70.5 ± 80.1	185.2 ± 78.6	−3.2	0.002
PNN20_1 Stage I and II	18.5 ±23.8	42.2 ± 18.7	–5.3	< 0.001
SD2 Stage I and II	126.3 ±146.8	61.54± 22.09	−5.9	< 0.001
LF_1 Stage I and II	151.5 ±155.6	574.3 ± 434.7	−3.6	< 0.001
SDNN Stage III and IV	30.6 ± 26.6	55.9 ± 33.8	−2.3	0.02
NN20 Stage III and IV	61.50±70.8	169.3±78.7	−4.0	< 0.001
PNN20 Stage III and IV	15.9 ±21.8	38.7 ± 18.7	−3.1	0.003
VLF_2 Stage III and IV	90.8 ± 117.3	269.4 ± 233.6	−2.7	0.01

### E. Association Between EEG PCRS and HRV

Finally, we examined the relations between EEG and ECG during VR induction. Five HRV indexes significantly correlated with the frequency-specific EEG PCRs in the healthy group (Table 4); MRRI was negatively associated while MHR was positively associated with $\alpha $ and $\beta $ frequency band PCRs at FCz and Pz. LF/HF were positively related with $\gamma $ PCRs at all channels during Stage III and IV. **SDNN** was related negatively with $\ominus $ FCz, $\beta $ FCz and $\gamma $ Pz during Stage III and IV. SD2 were negatively related with $\ominus $ Cz and $\ominus $ Pz during Stage I and II and $\ominus $ FCz, $\ominus $ Pz, $\alpha $ Pz, $\beta $ FCz, $\beta $ Pz and $\gamma $ Pz during Stage III and IV. Interestingly, no HRV indexes in the patient group were found to associate with the EEG PCRs.

**TABLE 4:** Significant correlations between EEG PCRS and HRV in the control group

EEG Features	SD2				MRRI		MHR		LF/HF		SDNN	
	Stage I & II		Stage III & IV		Stage III & IV		Stage III & IV		Stage III & IV		Stage III & IV	
	r [95% CI]	${P}$	r [95% CI]	${P}$	r [95% CI]	${P}$	r [95% CI]	${P}$	r [95% CI]	${P}$	r [95% CI]	${P}$
θ FCz			−0.6 [−0.8, −0.1]	0.021							−0.5 [−0.8, −0.1]	0.031
θ Cz	−0.5 [−0.8,-0.1]	0.032										
θ Pz	−0.5 [−0.8, 0]	0.045	−0.6 [−0.9, −0.2]	0.012								
$\alpha $FCz					−0.6 [−0.8, −0.1]	0.014	0.6 [0.2, - 0.8]	0.012				
$\alpha $Pz			−0.5 [−0.8, 0]	0.046	−0.6 [−0.9, −0.2]	0.007	0.6 [0.2, - 0.9]	0.007				
$\alpha $C4					−0.5 [−0.8, 0]	0.045	0.5 [0, 0.8]	0.034				
$\beta $FCz			−0.6 [−0.8, −0.1]	0.026	−0.6 [−0.8, −0.1]	0.023	0.6 [0.1, 0.8]	0.018			−0.5 [−0.8, 0]	0.035
$\beta $Pz			−0.6 [−0.8, −0.2]	0.013	−0.6 [−0.9, −0.2]	0.011						
$\gamma $C3									0.6 [0.1, 0.8]	0.023		
$\gamma $C4									0.6 [0.1, 0.8]	0.023		
$\gamma $FCz									0.6 [0.1, 0.8]	0.021		
$\gamma $Cz									0.6 [0.1, 0.8]	0.026		
$\gamma $Pz			−0.5 [−0.8, - 0]	0.04					0.6 [0.1, 0.8]	0.024	−0.5 [−0.8, 0]	0.048

## V. Discussion and Conclusion

In this study, we used an integrated VR system with gradual intensities of drug cues to investigate the EEG activities and ECG variabilities that may reflect different stimulations. We observed that this VR system induced brain changes in both groups, but only patients exhibited stimulation-specific neuronal differences. Furthermore, six HRV indexes significantly differed between the two groups and significant relations between HRV indexes and EEG changes were only identified in the healthy controls, not in MUD patients.

### A. Stage-Specific Neuronal Differences in Response to Drug Cues Only in Patients with Mud

Previous studies have reported that healthy controls did not show significant changes in response to METH cues compared to neutral cues, leading to the explanation that cue reactivity may indeed be a result of Pavlovian conditioning (see for [37] a review). This is based on the reasoning that no drug expectancies among non-drug users leads to lack of cue reactivity [37]. In this study, we found that only patients with MUD, not healthy controls, exhibited the stage-specific differences in response to drug cues during VR induction. Our finding further supports the Pavlovian conditioning of drug cues by showing that different drug cues are indiscriminate to healthy controls because of no related experience, but are meaningful to patients with MUD. Particularly, $\alpha $ and $\beta $ activities over the midline of the brain were significantly enhanced during the fourth stage. Finally, the finding that the two groups significantly differed only in Stage IV suggests the important role of stimulation intensity to differentiate the patients from healthy controls.

The functional roles of local $\alpha $ oscillations are thought to be associated with cognitive performance, memory, attention, and inhibitory controls of motor programs (see [38] for a review). Previous studies have shown that relatively higher $\alpha $ oscillations were associated with high impulsivity and trait anxiety [39] or with affective contents of the stimuli that have been memorized [40]. Additionally, the $\beta $ activity was reported to be engaged in active attention or/and motor preparation [27] and negatively associated with inhibiting the behavior that would lead to punishment [17]. In this study, $\alpha $ and $\beta $ PCRs in patients with MUD were significantly enhanced in the fourth stage, indicating a stronger level of intrinsic impulsivity and/or a higher affective association with the drug-related stimuli. In summary, patients with MUD exhibited abnormal $\alpha $ and $\beta $ PCRs in response to specific drug cues. The finding of drug cue-specific PCR changes may be used to separate MUD patients from healthy controls.

### B. HRV Abnormalities and Disruptions of Brain-Heart Relations in Patients With MUD

In this study, we observed that HRV indexes of MRRI, SDNN, NN20, PNN20, VLF, LF and SD2 significantly differed between patients and controls during VR induction. Previous studies reported the significant differences between patients and controls in SDNN, RMSSD, PNN50, LF, HF, LF/HF during VR exposure and SD2, NN20, and PNN20 after VR exposure to drug cues [18], [19]. Furthermore, Wang et al. demonstrated significant differences in HRV indexes on time (SDNN, pNN50, and RMSSD) and non-linear (SD1 and SD2) domains from baseline to follow-up assessments during exposure to VR cues [41]. Our findings are in line with previous reports that these HRV indexes significantly differed between the patient and control groups during VR drug-cue induction, suggesting HRV abnormalities in patients with MUD.

The frequency-related variabilities of LF and HF reflect the state changes of sympathetic and parasympathetic nerve systems, respectively, and LF/HF represents the active balance of the autonomic nerve system [42]. Ewing et al. reported that PNN50 is a reliable indicator of parasympathetic nervous system [43]. Therefore, our findings of HRV abnormalities in MUD patients indicate abnormal changes of the autonomic nerve system. Furthermore, we observed that only healthy controls had significant relations between HRV indexes and EEG changes. It has shown that the central nervous system can alter the autonomic nerve system, leading to declined task performance and decreased vagal nerve activity while increasing sympathetic nerve activity [44]. This brain-heart interaction was further supported by the experimental results of electrical stimulation at different auricular locations [45]. Machetanz et al. reported that parasympathetic (RMSSD, PNN50, MRRI) and sympathetic (SDNN) changes were related to frequency-specific oscillatory changes in different brain areas in healthy subjects during electrical stimulation of the vagus nerve [45]. Despite our experimental setup differing from the aforementioned studies, we also identified significant relations between HRV changes of SDNN, MRRI, and LF/HF and EEG changes only in healthy subjects. Taken together, these results indicate that patients with MUD exhibited not only HRV abnormalities but also a disruption of brain-heart relations.

### C. Study Considerations

In this study, we recruited 32 subjects- 16 patients and 16 healthy controls. This leads to the question whether the sample size is enough for finding true results. Indeed, an adequate sample size of a study is of importance to conclude statistical significance [46]. It was suggested that other statistical evaluations, for instance, effect size, should also be considered for decision making because traditional null hypothesis significance testing (NHST) is sensitive to sample size [47]. Previous studies reported that a good effect size (0.5~1.4) estimated using Cohen’s $d$ increases but a small effect size (0.01~0.2) decreases statistical power [48], [49]. Accordingly, a sample size of 16 is sufficient for data with good quality (i.e. the effect size > 0.5) [50], although most highly cited neuroimaging studies with patient participants (>1000+ citations) had median sample size of 14.5 [51]. We, therefore, examined the effect sizes based on the parameters under investigation, including EEG PCRs and HRV indexes, using 16 patients and 16 healthy controls (please see the supplementary table 2). Overall, 43.6% effect sizes were greater than 0.5 and the majority of effect sizes (73.1%) were greater than 0.3 (i.e. the moderate effect size), suggesting fairly good data quality and sample size in this study. However, this does not automatically prevent the probability of finding false results [52], [53], [54]. A large-scale trial is needed to further confirm these findings of small sample size in the present study.

In conclusion, we found that only patients with MUD exhibited stimulation-specific neuronal differences when using our VR system. Furthermore, six HRV indexes significantly differ between the two groups and significant relations between HRV indexes and EEG changes were identified only in the healthy controls, not in MUD patients. Nevertheless, our findings of stimulus-specific EEG changes and the impaired EEG-ECG relations in patients with MUD shed light on the understanding of drug-cue reactivity and may be used to design diagnostic and/or therapeutic strategies for MUD.
